# Safety and efficacy of aspirin after combined cerebral revascularization for ischemic moyamoya disease: A prospective study

**DOI:** 10.3389/fsurg.2023.1091062

**Published:** 2023-05-24

**Authors:** Bingqian Xue, Shao Zhang, Gaochao Guo, Ruiyu Wu, Kaiwen Gu, Liming Zhao, Chaoyue Li

**Affiliations:** ^1^ Department of Neurosurgery, Henan University People's Hospital, Zhengzhou, China; ^2^ Department of Neurosurgery, Henan Provincial People's Hospital, Zhengzhou, China; ^3^ Department of Neurosurgery, First Affiliated Hospital of Henan University, Kaifeng, China; ^4^ Department of Neurosurgery, Zhengzhou University People's Hospital, Zhengzhou, China

**Keywords:** moyamoya disease, aspirin, STA-MCA-EDMS, bypass efficacy, postoperative complications

## Abstract

**Objective:**

To analyze the safety and efficacy of regular aspirin use after combined cerebral revascularization in patients with ischemic moyamoya disease.

**Methods:**

From December 2020 to October 2021, a total of 326 patients diagnosed with ischemic moyamoya disease by global cerebral angiography and undergoing first-time combined cerebral revascularization at the Moyamoya Disease Diagnosis and Treatment Research Center of our hospital were selected. Combined cerebral revascularization: superficial temporal artery-middle cerebral artery (STA-MCA) +encephalo-duro-myo-synangiosis (EDMS).Patients were screened by 2 senior physicians according to established inclusion/exclusion criteria. Patients were divided into aspirin and non-aspirin groups based on whether they received regular oral aspirin after surgery. A total of 133 patients were enrolled in the aspirin group. A total of 71 patients (204 cases) were enrolled in the non-aspirin group. Related data were collected before and 1 year after surgery and statistically analyzed to assess the prognosis of both groups.

**Results:**

In the two groups, the mRS Score was significantly different after one year (*P* = 0.023). TIA occurred in 26 patients (19.5%) in the aspirin group and 27 patients (38.0%) in the non-aspirin group within one year after surgery, and the difference between the two groups was statistically significant (*P* = 0.004). There was no significant difference in cerebral perfusion stage, the improvement rate of cerebral perfusion, Matsushima grading, bypass patency, and other complications within one year after the operation (*P* > 0.05).

**Conclusions:**

In patients with ischemic moyamoya disease who underwent combined cerebral revascularization, postoperative administration of aspirin can reduce the incidence of TIA without increasing the risk of bleeding, but it can not significantly improve the cerebral perfusion of the operation side, Matsushima grading, and bypass patency.

## Introduction

Cerebral revascularization is the main treatment for moyamoya disease, which can effectively improve cerebral perfusion, prevent stroke and improve long-term neurological function. Surgical treatment is also divided into direct revascularization, indirect revascularization, and combined revascularization based on the characteristics of different regions. The 2021 guidelines for the management of moyamoya disease in Japan (1) pointed out that indirect cerebral revascularization alone is not sufficient for adult patients with moyamoya disease, and direct or combined cerebral revascularization surgery is needed to improve cerebral perfusion. Adequate control of blood pressure and fluid infusions, maintenance of normocapnia, and anti-platelet therapy are required during the perioperative period to avoid complications of cerebrovascular events on the operated side and the contralateral side. There is currently no consensus on long-term medication after surgery. Studies have shown that intravascular microthromboembolism may be one of the causes in patients with ischemic moyamoya disease, therefore the use of antiplatelet drugs is increasing (2–4). However, there is no consensus on the timing and duration of aspirin use in patients with moyamoya disease, and the results of published studies are also different. This study aims to evaluate the efficacy and safety of aspirin in ischemic moyamoya disease after combined cerebral revascularization by neurological function score and imaging examination.

## Materials and methods

### Inclusion criteria and exclusion criteria

**Figure 1:**
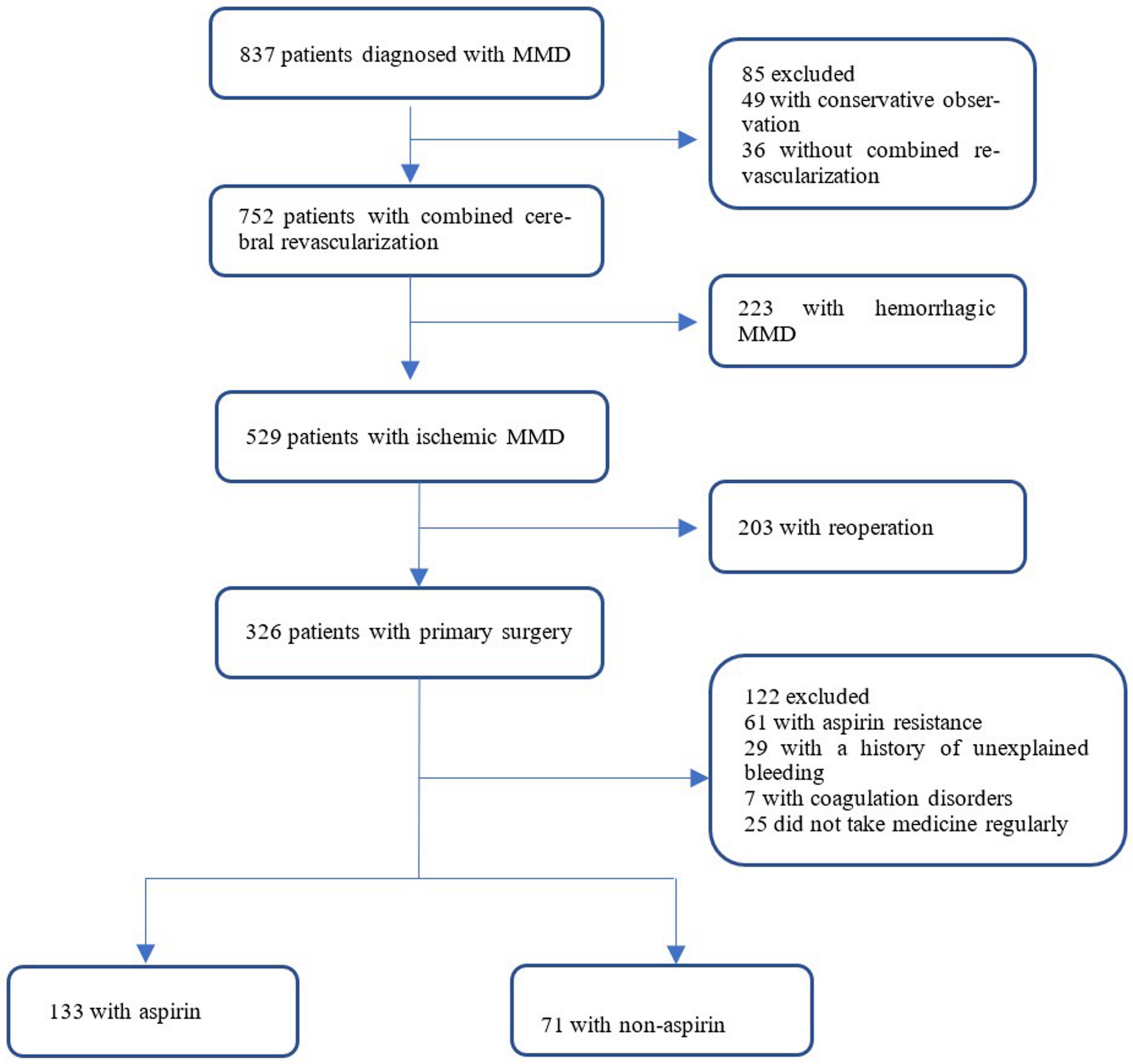
Flow diagram of the study population. MMD, Moyamoya disease.

A total of 326 patients were diagnosed with ischemic moyamoya disease by cerebral angiography and magnetic resonance imaging and underwent combined cerebral revascularization for the first time in the Moyamoya Disease Diagnosis and Treatment Center, Cerebrovascular Disease Hospital, Henan Provincial People's Hospital from December 2020 to October 2021 were enrolled. Patients were screened by two senior physicians according to established inclusion/exclusion criteria and were divided into two groups based on whether they took aspirin regularly after surgery. 133 patients were enrolled in the aspirin group. 71 patients were included in the non-aspirin group (Figure 1).

Inclusion criteria: (1) Patients diagnosed with moyamoya disease according to the 2019 Chinese Expert Consensus on the Treatment of Moyamoya Disease(Chinese expert consensus group on the treatment of moyamoya disease) (5); (2) Previous symptoms of cerebral ischemia include dizziness and headache, reversible neurological deficits, TIA, limb movement disorders, cerebral infarction, and seizures; (3) The patient underwent the first combination of cerebral revascularization at our hospital: superficial temporal artery-to-middle cerebral artery (STA-MCA) bypass and encepho-dural-myo-synangiosis (EDMS) bypass; (4) Patients voluntarily participated in this study and signed informed consent. Exclusion criteria: (1) Patients with aspirin resistance; (2) Patients had a history of unexplained bleeding; (3) Patients have haemophilia or thrombocytopenia or other clotting disorders; (4) Patients' compliance was poor and medication was irregular.

### The general information

In the non-aspirin group, there were 40 male patients (56.3%) and 31 female patients (43.7%). Among the 133 patients in the aspirin group, 68 (51.1%) were male and 65 (48.9%) were female. The age of the non-aspirin group was 48 (34,50) years old. The age of patients in the aspirin group was 47 (37,54) years old. In the non-aspirin group, there were 9 cases (12.7%) in Suzuki stage 2, 36 cases (50.7%) in Suzuki stage 3, 20 cases (28.2%) in Suzuki stage 4, and 6 cases (8.5%) in Suzuki stage 5. In the aspirin group, there were 21 cases (15.8%) in Suzuki stage 2, 61 cases (45.9%) in Suzuki stage 3, 43 cases (32.3%) in Suzuki stage 4, and 8 cases (6.0%) in Suzuki stage 5.

### Methods of perioperative assessment and follow-up

Baseline information such as age, sex, first symptoms, family history of stroke, mRS score, Suzuki stage, cerebral perfusion stage, and other data was collected from both groups before surgery. The modified Rankin scale (mRS) was evaluated at discharge. Patients were followed up at 3, 6, and 12 months after surgery to evaluate mRS and short-term complications (TIA, cerebral infarction, cerebral hemorrhage, epilepsy). The first imaging follow-up is done within 6–12 months of the surgery.DSA and PWI were rechecked to assess Matsushima grading, bypass patency, and cerebral perfusion improvement.

### Observation indicators

**Figure 2:**
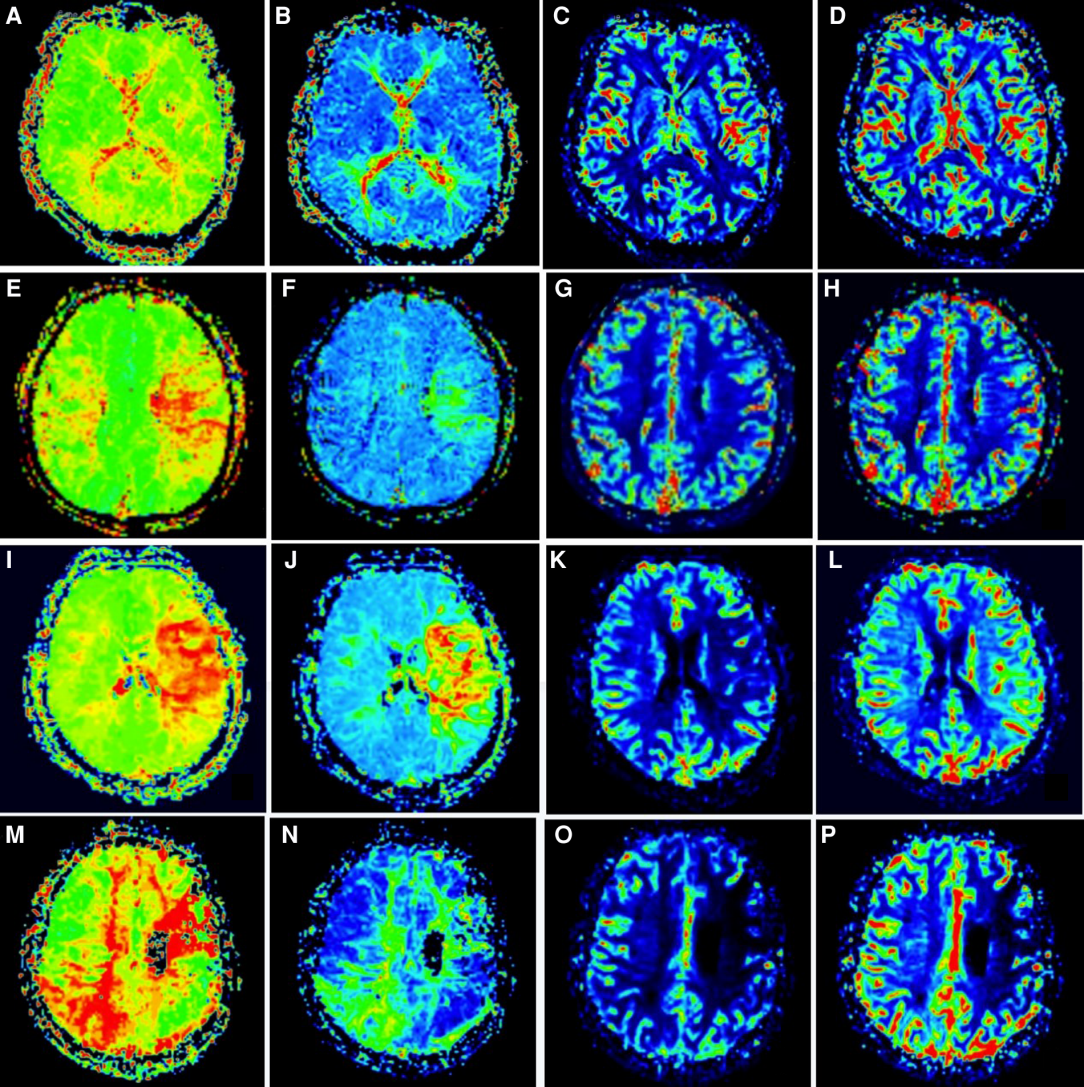
Staging of cerebral perfusion (left) Figures A-D shows stage I. Figure E-H shows stage II; Figure I-L shows stage III; Figure M-P shows stage IV.

(1) Efficacy evaluation: according to the severity of symptoms, the patients with mRS Score of 0–1 were classified into the good group, 2–3 were classified into the general group, and 4–5 were classified into the severe group. Postoperative complications were defined as the occurrence of TIA, cerebral hemorrhage, cerebral infarction, epilepsy, and other neurological deficits from the time of surgery to the last follow-up.
(2) Imaging indicators: According to the global cerebral angiography results of the patients half a year after the first combined cerebral revascularization, the postoperative neoangiogenesis was divided into three groups according to the criteria proposed by Matsushima (6), which was the proportion of the distribution area of the middle cerebral artery provided by surgical neoangiogenesis to evaluate the neoangiogenesis of the patients: Grade A, more than 2/3 of the middle cerebral artery territory was covered by neovascularization. Grade B, 1/3 to 2/3 of the middle cerebral artery supply area is covered by neoangiogenesis. Grade C, less than 1/3 of the middle cerebral artery territory was covered by neoangiogenesis, or no neovascularization was observed. During PWI, the cerebellum was considered a region of interest for comparative study. According to the cerebral perfusion study of Gao (7), the hypoperfusion state before cerebral infarction was divided into four stages: stage I, prolonged TTP, normal MTT, rCBF, and rCBV; In stage II, TTP and MTT were prolonged, rCBF was normal, and rCBV was normal or slightly increased. In stage III, TTP and MTT were prolonged, rCBF was decreased, and rCBV was essentially normal or slightly decreased. TTP and MTT were prolonged, and rCBF and rCBV were decreased in stage IV (Figure 2). A decrease in the cerebral perfusion stage was considered an improvement, and an increase was considered a deterioration.

### Statistical analysis

All analyses were performed using IBM SPSS statistical software V.26.0 (IBM). 95% CI was *P* < 0.05, which was statistically significant. Measurements of non-normal distribution are expressed by the median (quartile range)M (P25-P75). T-test was used for continuous variables and *χ*^2^ test for categorical variables. The association between postoperative aspirin therapy and complication outcome was expressed as hazard ratio (RRs) and 95% CI, and *χ*^2^ was used to calculate the risk ratio (RRs) and 95% CI, and the statistical significance of RR was tested.

## Results

A total of 204 patients were enrolled in the study. Baseline characteristics did not differ significantly between patients taking aspirin (aspirin group, 100 mg/day) and patients not taking aspirin (non-aspirin group) in terms of age, sex, medical history, smoking and drinking status, preoperative MRS score, Suzuki stage, and preoperative cerebral perfusion stage (*P* > 0.05) (Table 1).

**Table 1:** Baseline characteristics of patients in the aspirin and non-aspirin groups.

Characteristic	Non-aspirin (*n* = 71)	Aspirin (*n* = 133)
**Age, years, mean (SD)**	48 (34, 50)	47 (37.5, 54)
**Gender, *n* (%)**		
Male	40 (56.3)	68 (51.1)
Female	31 (43.7)	65 (48.9)
**Symptoms, *n* (%)**		
TIA	41 (57.8)	68 (51.1)
Cerebral infarction	16 (22.5)	35 (26.3)
Others	14 (19.7)	30 (22.6)
**Diabetes, *n* (%)**	7 (9.9)	15 (11.3)
**Hypertension, *n* (%)**	21 (29.6)	47 (35.3)
**Hyperlipidemia, *n* (%)**	17 (0.24)	29 (21.8)
**Smoking history, *n* (%)**	22 (31.0)	32 (24.1)
**Drinking history, *n* (%)**	13 (18.3)	25 (18.9)
**Family history of cerebrovascular disease, *n* (%)**	14 (19.7)	26 (19.6)
**mRS at admission, *n* (%)**		
0–1	39 (54.9)	87 (65.4)
2–3	29 (40.8)	39 (29.3)
4–5	3 (4.2)	7 (5.3)
**Suzuki stage, *n* (%)**		
1	–	–
2	9 (12.7)	21 (15.8)
3	36 (50.7)	61 (45.9)
4	20 (28.2)	43 (32.3)
5	6 (8.5)	8 (6.0)
6	–	–
**Staging of cerebral perfusion at admission, *n* (%)**		
1	7 (9.9)	11 (8.3)
2	19 (26.8)	42 (31.6)
3	23 (32.4)	45 (33.8)
4	22 (30.9)	35 (26.3)
**Operation side, *n* (%)**		
Left	41 (57.7)	84 (63.2)
Right	30 (42.3)	49 (36.8)

SD, standard deviation; TIA, transient ischemic attack; mRS, modified Rankin scale.

### Improvement in clinical symptoms

In the comparison of mRS Scores between the two groups at 1 year after surgery, 114 patients (85.7%) had 0–1 score, 14 patients (10.5%) had 2–3 score, and 5 patients (3.8%) had 4–5 score in the aspirin group. In the non-aspirin group, 51 patients (71.8%) had 0–1 score, 18 patients (25.4%) had 2–3 score, and 2 patients (2.8%) had 4–5 score, and the difference was statistically significant (*Z* = −2.275, *P* < 0.05) (Table 2).

**Table 2:** Bypass efficacy in patients with aspirin or non-aspirin therapy.

Outcome	Non-aspirin (*n* = 71)	Aspirin (*n* = 133)	*Z*	*P*
**Follow-up mRS, *n* (%)**			−2.275	0.023
0–1	51 (71.8)	114 (85.7)		
2–3	18 (25.4)	14 (10.5)		
4–5	2 (2.8)	5 (3.8)		
**Follow-up staging of cerebral perfusion, *n* (%)**			−0.316	0.752
I	12 (16.9)	29 (21.8)		
II	28 (39.4)	47 (35.3)		
III	21 (29.6)	36 (27.1)		
IV	10 (14.1)	21 (15.8)		
**Follow-up improvement rate of cerebral perfusion, *n* (%)**			−1.201	0.230
Improve	48 (67.6)	102 (76.7)		
No Change	17 (23.9)	18 (13.5)		
Deteriorate	6 (8.5)	13 (9.8)		
**Follow-up Matsushima stage, *n* (%)**			−1.365	0.172
A	11 (15.5)	20 (15.0)		
B	29 (40.8)	72 (54.1)		
C	31 (43.7)	41 (30.8)		
**Bypass patency, *n* (%)**			−0.928	0.335
Yes	59 (83.1)	117 (88.0)		
No	12 (16.9)	16 (12.0)		

### Imaging evaluation

In the non-aspirin group, 12 patients (16.9%) had cerebral perfusion stage I, 28 patients (39.4%) had cerebral perfusion stage II, 21 patients (29.6%) had cerebral perfusion stage III, and 10 patients (14.1%) had cerebral perfusion stage IV. In the aspirin group, there were 29 cases (21.8%) with cerebral perfusion stage I, 47 cases (35.3%) with cerebral perfusion stage II, 36 cases (27.1%) with cerebral perfusion stage III, and 21 cases (15.8%) with cerebral perfusion stage IV. There were 48 cases (67.6%) with cerebral perfusion improvement in the non-aspirin group and 102 cases (76.7%) with cerebral perfusion improvement in the aspirin group, and the difference was not statistically significant (*P* > 0.05). There were 11 cases (15.5%) of Matsushima grading A, 29 cases (40.8%) of grade B, and 31 cases (43.7%) of grade C in the Non-aspirin group. In the aspirin group, 20 cases (15.0%) were grade A. There were 72 cases (54.1%) of grade B and 41 cases (30.8%) of grade C. There were 59 cases (83.1%) of bypass patency in the non-aspirin group and 117 cases (88.0%) in the aspirin group, and the difference was not statistically significant (*P* > 0.05) (Table 2).

### Postoperative complications

There were 27 cases (38.0%) of TIA in the non-aspirin group and 26 cases (19.5%) of TIA in the aspirin group within 1 year after the operation, and the difference between the two groups was statistically significant (RR: 0.51, 95% CI: 0.33–0.81; *P* = 0.004). There were 4 cases (5.6%) of recurrent cerebral infarction, 2 cases (2.8%) of cerebral hemorrhage, and 2 cases (2.8%) of epilepsy in the Non-aspirin group. In the aspirin group, there were 7 cases (5.3%) of recurrent cerebral infarction, 3 cases (2.3%) of cerebral hemorrhage, and 2 cases (1.5%) of epilepsy. There was no significant difference between the two groups (*P* > 0.05) (Table 3).

**Table 3:** Risk of ischemic complications and hemorrhagic events with postoperative aspirin therapy.

Outcome	Non-aspirin	Aspirin	RR (95%CI)	*P*
TIA, *n* (%)	27 (38.0)	26 (19.5)	0.51 (0.33–0.81)	0.004
Ischemic stroke, *n* (%)	4 (5.6)	7 (5.3)	0.93 (0.28–3.08)	0.911
Hemorrhagic stroke, *n* (%)	2 (2.8)	3 (2.3)	0.80 (0.14–4.68)	0.805
Seizure, *n* (%)	2 (2.8)	2 (1.5)	0.53 (0.08–3.71)	0.519

RR, relative risk; CI, confidence interval.

## Discussion

This study aims to analyze whether regular oral aspirin (100 mg/day) can further improve the prognosis of patients with ischemic moyamoya disease after combined cerebral revascularization. The findings suggest that aspirin is effective in improving patients' symptoms and reducing the incidence of TIA.

Several studies have shown that antiplatelet therapy can improve the prognosis of patients with cerebral infarction (8, 9). Although in addition to aspirin, additional antiplatelet drugs such as clopidogrel or cilostazol are widely used for stroke prevention, aspirin is still the preferred antiplatelet drug for the treatment of moyamoya disease in most studie (10). A European survey in 2021 found that long-term conservative treatment with aspirin in some patients with confirmed ischemic type improved mRS Scores to a certain extent, which was in excellent consistency with the surgical treatment group (11). However, Pang showed that antiplatelet drugs could not reduce the risk of cerebral infarction in patients with moyamoya disease treated conservatively within a follow-up period of no less than 6 months, nor could they improve the ischemic symptoms of patients (12). A Japanese national survey showed that 52.5% of the surveyed units supported the use of antiplatelet drugs for a period of time after moyamoya disease surgery (10). A recent meta-analysis showed that aspirin administration within 48 h of stroke onset reduced the risk of early recurrent ischemic stroke without increasing the risk of bleeding and improved long-term outcomes (13). We also observed that in cerebral ischemic patients with moyamoya disease within 1 year after blood supply reconstruction regular oral aspirin mRS score improved more significantly (*P* = 0.023). The likely reason is that aspirin has an anti-platelet effect, and not only does it have an obvious anti-inflammatory analgesic effect, but the first symptoms such as headache and dizziness are somewhat reduced. However, there were no significant differences between the two groups in the rates of bypass patency and improvement in cerebral perfusion at 6 months after surgery (*P* > 0.05), and we did not observe an increase in the risk of bleeding. Lu et al. showed that after STA-MCA bypass surgery for ischemic moyamoya disease, the bypass patency of the oral aspirin group was significantly higher than that of the non-aspirin group (98.7% vs. 89.7%; *P* = 0.012) (14). Our results did not show a significant between-group difference in bypass patency at 1 year (*P* = 0.335), and we did not observe an increase in the risk of bleeding. The reason for the lower overall patency rate than in the Lu study is the use of combined cerebral revascularization in our study, and there may be competitive inhibition between the two procedures, which may explain the effectiveness of indirect neoangiogenesis. Zhao showed that found that the bypass occlusion rate after direct cerebral revascularization was 23.5%, and the occlusion rate after combined cerebral revascularization was 26.7%, which is worthy of further study (15). The results of this study show that aspirin use after combined cerebral revascularization in ischemic moyamoya disease is beneficial only in alleviating symptoms and reducing TIA attacks, but has no significant effect on improving postoperative cerebral blood perfusion, promoting collateral circulation, maintaining bypass patency, and reducing the incidence of stroke. Therefore, aspirin therapy is recommended in patients with frequent postoperative TIA.

## Limitation

There are still some limitations to this study. First, in this study, two senior physicians selected subjects strictly according to the inclusion and exclusion criteria to avoid selection bias to the greatest extent, but it could not completely avoid the problem. Secondly, the number of patients included in this study was small and the follow-up was only 1 year, so the long-term prognosis could not be determined. Future multi-center prospective studies are needed to verify its effectiveness.

## Data Availability

The original contributions presented in the study are included in the article/supplementary material, further inquiries can be directed to the corresponding author.
